# The challenge of equipoise in trials with a surgical and non-surgical comparison: a qualitative synthesis using meta-ethnography

**DOI:** 10.1186/s13063-021-05403-5

**Published:** 2021-10-07

**Authors:** Loretta Davies, David Beard, Jonathan A. Cook, Andrew Price, Ida Osbeck, Francine Toye

**Affiliations:** 1 Nuffield Department of Orthopaedics, Rheumatology and Musculoskeletal Sciences, Botnar Research Centre, University of Oxford, Headington, Oxford, OX3 7LD UK; 2 Lund University, Lund, Sweden; 3 Nuffield Orthopaedic Centre, Oxford University Hospitals NHS Foundation Trust, Oxford, UK

**Keywords:** Qualitative evidence synthesis, Surgical interventions, Non-surgical interventions, Recruitment challenges

## Abstract

**Background:**

Randomised controlled trials in surgery can be a challenge to design and conduct, especially when including a non-surgical comparison. As few as half of initiated surgical trials reach their recruitment target, and failure to recruit is cited as the most frequent reason for premature closure of surgical RCTs. The aim of this qualitative evidence synthesis was to identify and synthesise findings from qualitative studies exploring the challenges in the design and conduct of trials directly comparing surgical and non-surgical interventions.

**Methods:**

A qualitative evidence synthesis using meta-ethnography was conducted. Six electronic bibliographic databases (Medline, Central, Cinahl, Embase and PsycInfo) were searched up to the end of February 2018. Studies that explored patients’ and health care professionals’ experiences regarding participating in RCTs with a surgical and non-surgical comparison were included. The GRADE-CERQual framework was used to assess confidence in review findings.

**Results:**

In total, 3697 abstracts and 49 full texts were screened and 26 published studies reporting experiences of patients and healthcare professionals were included. The focus of the studies (24/26) was primarily related to the challenge of recruitment. Two studies explored reasons for non-compliance to treatment allocation following randomisation. Five themes related to the challenges to these types of trials were identified: (1) radical choice between treatments; (2) patients’ discomfort with randomisation: I want the best treatment for me as an individual; (3) challenge of equipoise: patients’ a priori preferences for treatment; (4) challenge of equipoise: clinicians’ a priori preferences for treatment and (5) imbalanced presentation of interventions.

**Conclusion:**

The marked dichotomy between the surgical and non-surgical interventions was highlighted in this review as making recruitment to these types of trials particularly challenging. This review identified factors that increase our understanding of why patients and clinicians may find equipoise more challenging in these types of trials compared to other trial comparisons. Trialists may wish to consider exploring the balance of potential factors influencing patient and clinician preferences towards treatments before they start recruitment, to enable issues specific to a particular trial to be identified and addressed. This may enable trial teams to make more efficient considered design choices and benefit the delivery of such trials.

**Supplementary Information:**

The online version contains supplementary material available at 10.1186/s13063-021-05403-5.

## Background

Surgical trials are considered challenging to design and conduct with only around 50% of initiated surgical trials reaching their original recruitment target [1–3]. Recruitment difficulties are cited as the most frequent reason for premature closure of surgical randomised controlled trials (RCTs) [4]. Difficulties with recruitment are especially evident when the interventions being evaluated are markedly different, such as trials comparing surgical and non-surgical interventions [5–7]. Low recruitment not only has implications for internal and external validity but may also lead to extended recruitment periods with implications for finance and resources. The potential also exists for delaying the introduction of effective treatment. For trials evaluating interventions already embedded in routine practice, delays in reporting could also have implications for continued inappropriate use of resources and suboptimal care [8]. Substantial amounts of public funds are invested in medical research; for example in 2018/2019, the National Institute of Health Research (NIHR) spent £317 million over their broad range or research programmes [9]. Given these potential negative consequences of incomplete RCTs, improving the design and conduct of such trials evaluating surgery and minimising the risk of failure should be a priority [7].

Qualitative research has been used to understand challenges in the conduct of difficult randomised controlled trials (RCTs), such as those in surgery, and inform the development of strategies to improve the design and conduct of trials [10–12]. Syntheses of qualitative studies, such as meta-ethnographies, can increase our understanding of the complex process of heath care and improve the experience and quality of care. They can also provide evidence of the acceptability, feasibility and appropriateness of interventions or services [13, 14] and can illuminate people’s experience of illness and healthcare [15].

Several reviewers have applied synthesised qualitative research to further understand the challenges of trial recruitment [11, 16–19] and have provided insights into the complexities of recruitment. For example, strong preferences for treatment of both patients and clinicians can pose a challenge to recruitment [11, 17, 19]. In addition, communication of trial information and significant trial components may influence potential participants’ decision to participate [11, 17–19].

The marked differences between the surgical and non-surgical interventions evaluated in these types of trial comparison are likely to contribute to the complexities of recruitment. Given the difficulties with recruitment are especially evident in trials directly comparing surgery and non-surgical interventions [5–7], understanding potential challenges may help to improve the design and conduct of trials of this specific type of comparison. At the present time, no review has been conducted to understand the specific challenges of conducting trials with surgical and non-surgical intervention comparators. The aim of this review was to conduct a qualitative evidence synthesis of studies that explored the experiences of patients and healthcare professionals participating in trials comparing surgical and non-surgical interventions and to identify challenges to their design and conduct.

## Methods

A qualitative evidence synthesis using meta-ethnography [20] was conducted. This interpretative approach to knowledge synthesis, proposed by Noblit and Hare [20], was considered the most appropriate method for this review to enable the development of a new conceptual understanding of the primary studies, whilst preserving the interpretative properties of the primary data [21].

Noblit and Hare [20] describe a seven-step process to meta-ethnography, which start with formulating a research idea through to expressing the findings of the research (Table 1). The stages outlined are not discrete but form part of an iterative research process [20, 21]. The analysis involves a process of identifying the key ideas or “concepts” from the primary qualitative studies, abstracting these concepts into conceptual categories, further abstracting categories into themes and finally developing a line of argument. The line of argument refers to building up a picture of all the aspects of the synthesised parts using a short paragraph, diagram or conceptual model [21]. Central to this approach of synthesising qualitative research is developing a conceptual understanding through the process of constant comparison and abstraction rather than alternative approaches which describe the findings [20].

**Table 1:** Seven steps of Noblit and Hare’s meta-ethnography [20]

	Seven steps of Noblit and Hare’s meta-ethnography (Noblit, 1988)
1.	**Getting started**
2.	**Deciding what is relevant**
3.	**Reading the studies**
4.	**Determining how the studies are related**
5.	**Translating the studies into one another**
6.	**Synthesising translations**
7.	**Expressing the synthesis**

### Phase 1: Selecting meta-ethnography and getting started

The review team were aware of a growing body of qualitative research exploring the challenges in the conduct of randomised trials [22]. We were particularly interested in understanding the factors that may contribute to difficulties in conducting trials with a surgical and non-surgical comparison, specifically in the perceptions and experiences of patients and healthcare professionals.

Our initial literature search identified a number of qualitative reviews designed to further understand the challenges of trial recruitment in general [11, 16–19]. These reviews have provided valuable insights and identified important factors, such as strong patient and clinician references for treatment, that may contribute to the complexities of recruitment. However, there were no qualitative syntheses specific to trials of surgery versus a non-surgical comparison.

We believed that synthesising the views, attitudes and experiences of both patients and clinicians participating in this type of trial comparison would enable us to further understand the specific complexities of conducting trials with this a surgical and non-surgical comparison, which are known to be particularly challenging to conduct [5–7, 20, 21, 23].

### Phase 2: Deciding what is relevant

This stage of the review involved systematically searching, screening and appraising potential studies to decide which to include in the synthesis.

Consensus on whether the search needs to include all available studies to undertake a ‘good’ qualitative synthesis has not be reached [24, 25]. In their original text on meta-ethnography, Noblit and Hare [20] do not advocate an extensive literature search when conducting a review. Unlike a quantitative review, a qualitative synthesis does not aim to summarise the available body of evidence but to provide a conceptual and interpretative contribution. Although the number of qualitative studies conducted alongside clinical trials is increasing, studies in surgical trials compared with other areas such as behavioural trials remain rare [22]. A systematic search of the literature was therefore conducted, to be able to identify the studies published in the area and identify any gaps in knowledge. This approach has been successfully conducted with other reviews [15, 26].

Published reports of qualitative studies that explored experiences and behaviours of patients and healthcare professionals participating in RCTs with a surgical and non-surgical comparison were included. Details of the inclusion and exclusion criteria are included in Table 2. Perspectives of both patients and clinicians were included. Pilot, feasibility and mixed methods studies that included a qualitative method of data collection and analysis, where the qualitative component was clearly identifiable and could be extracted, were also included. Studies of paediatric trials were excluded because of the potential for challenges related only to the specific nature of the population being identified. Only articles in English were reviewed because of limited resources to provide translation services.

**Table 2:** Study inclusion and exclusion criteria using modified PICO [27, 28]

Inclusion	Exclusion
**Patient/Population:** Peer reviewed journal articles and conference papers published anytime up to end of February 2018. Patients or health care professionals (clinicians) stakeholder views participating in RCTs with a surgical and non-surgical comparison. Articles in English language published in any country.	Unpublished dissertations, book chapters, papers or conference abstracts without corresponding full text articles. Studies with participants under 18 years of age.
**Intervention:** RCTs with a surgical and non-surgical comparison	
**Comparison:** Randomised controlled trials that have a surgical and non-surgical comparison, e.g. physiotherapy, drugs, medical management.	
**Outcomes:** Challenges related to the design and conduct of RCTs with a surgical and non-surgical comparison such as recruitment, retention, compliance with treatment allocation.	
**Studies:** Qualitative studies (or mixed methods studies containing substantial qualitative components that can make a contribution to the meta-synthesis). As an operational definition data collected were in the form of semi-structured interviews, focus groups, open ended evaluation forms including free text responses, observational field notes, or reflective journals. Papers should report some form of thematic or inductive analysis.	Studies of recruitment into surgical studies that are not randomised controlled trials. No qualitative analysis undertaken, or primarily quantitative data reported. This includes questionnaire data.

### Search process

Searching for qualitative research related to RCTs can be challenging because it may be embedded in the wider trial making it difficult to identify [27]. Therefore, an iterative approach was taken combining several strategies.

To help develop the search strategy, an initial search for any existing qualitative evidence synthesis related to challenges in conducting clinical trials was conducted using terms developed by Booth [27].

The search terms from existing qualitative evidence synthesis in this area [11, 17] were reviewed to help inform search strategy for the synthesis is detailed in the Supplementary Appendix. The following databases were searched up to the end of February, 2018: Medical Literature Analysis and Retrieval System Online (MEDLINE), and the Cochrane Central Register of Controlled Trials (CENTRAL), Cochrane Methodology Register, CINAHL, Embase and PsycINFO. Electronic bibliographic database searches used a combination of medical subject headings (MESH) and free text. To increase the likelihood of identifying all suitable qualitative studies and validate the search strategy, the reference lists of included studies and the qualitative evidence synthesis identified in the initial search [11, 18, 29] were also searched for further potential studies.

### Selecting primary studies

One reviewer (LD) screened the titles and abstracts and articles that did not meet the inclusion criteria were excluded. Where it was unclear from the title and abstract whether the paper should be included, the full text articles were then reviewed and discussed with a researcher (co-author FT) with experience in qualitative evidence synthesis (QES), to reach agreement on which papers to include.

### Quality appraisal

Despite ongoing debate about how, or whether, to assess the quality of qualitative research [30–32], a growing number of researchers are appraising studies for the purpose of QES [33]. There are now many suggested frameworks for appraising the quality of qualitative research, although there is no consensus on what makes a study ‘good’ [30–32].

Quality appraisal of the studies was carried out by two reviewers (LD and IO) using the Critical Appraisal Skills Programme (CASP) checklist [34] (see Supplementary Appendix). Individual assessments were compared, and any areas of discrepancy were discussed with a researcher (co-author FT), to reach agreement. A decision was made in advance not to exclude studies with low-quality scores as it is recognised that studies deemed to be of low quality may still provide new insights [21]. We did however use the quality rating of the studies as one of the components in assessing our confidence in the findings from the review [35] (Supplementary Appendix: Table 5).

### Phases 3 and 4: Reading included studies and determining how studies are related

During this phase, two reviewers (LD and IO) read each paper thoroughly to become familiar with the studies and begin to identify concepts within the studies. This is not a discrete stage of the process as reading continues throughout. A concept is defined as ‘having some analytical or conceptual power’, unlike more descriptive themes [23]. The term ‘concept’ is used in this case to distinguish between the level of progressive abstraction of data and the original primary studies to develop the findings of the meta-ethnography.

The findings from the primary studies are the raw data of the meta-ethnography. The definition of first-, second- and third-order constructs by Schutz [36] are often used in meta-ethnography studies to distinguish the data used [37]. First-order constructs are defined as participants ‘common sense’ interpretations in their own words (participant quotations). Whereas second-order constructs are the primary researchers’ interpretations of the first-order constructs. In meta-ethnography, the ‘data’ are second-order constructs which are further abstracted to develop third-order constructs (reviewers’ interpretations of second-order constructs) which are the themes and findings of the QES.

The next step involved extracting the ‘raw data’ (first- and second-order constructs) from the primary studies for the synthesis. All full text articles were imported into NVivo 11 (QSR International, Warrington, UK) [38], the computer software used to facilitate qualitative data management. Using this software, all concepts relating to challenges of conducting surgical versus non-surgical trials (phenomenon of interest) identified within the original papers were coded by one reviewer (LD). The data was extracted verbatim so that there was no risk of losing important data and to preserve the original terminology used by the primary authors [37]. The category labels for the concepts created at this stage were descriptive, and the third-order constructs were developed during the next two phases. Two reviewers (LD and IO) discussed the concepts and in cases where it was agreed that there was no clear concept articulated, i.e. ‘no central idea pulling the description together’ [25], in the original source material, the data was excluded (although not necessarily the entire study).

Data on study characteristics was also extracted at this stage: setting, aim, sample and methods of data collected and analysis. This provided contextual information to allow the reviewers and readers to examine the relevance of each study and to determine how each study was related to each other and the overall study aims.

### Phases 5 and 6: Translating studies and synthesising translations

Translating studies into each other involves determining the relationship among the concepts from across the studies through constantly comparing and discussing concepts with a second reviewer (LD and IO).

Concepts from all included studies coded in NVivo were then added to an Excel spreadsheet table. From this table, the concepts from the different primary studies were discussed (LD and IO) and then clustered into relevant categories, grouped by the common concepts from the studies. An example of how, using thematic maps, concepts were compared to identify similarities and differences between them and organise them into further abstracted conceptual categories with shared meaning is shown in the Supplementary Appendix. The data was gradually organised into conceptual categories that reflected the key ideas or concepts. These newly formed conceptual categories were labelled using terminology which encompassed all the relevant concepts they contained. The conceptual categories continued to be compared and discussed by two reviewers (LD and IO) to develop the final themes. Once descriptions of the conceptual categories were agreed, they were then discussed with the wider team (LD, IO, DB and FT) and refined into final themes.

### Phase 7: Expressing the synthesis

The final stage of a meta-ethnography involves expressing the synthesis findings. The line-of-argument approach [20] was utilised to build a picture and integrate the themes to derive new insights to help us understand the challenges of conducting trials with a surgical versus non-surgical comparison. Four reviewers (LD, FT, JC, DB) discussed the abstracted themes and developed a model that integrated findings to give a new “storyline” or overarching explanation of the phenomenon [39]. A model was constructed to develop and refine the line of argument that reflected the final interpretation. The findings were also summarised, using the GRADE-CERqual approach to determining confidence in review findings, in the Summary of Qualitative Findings (SoQF) table [35].

The ‘eMERGe’ meta-ethnography reporting guidance was used to guide this report [40].

### Confidence in review findings—GRADE-CERQual

The ‘Confidence in the Evidence from Reviews of Qualitative research’ (GRADE-CERQual) approach has been developed to provide a structured method for assessing confidence in the evidence from a QES, and this framework used for this review [41]. GRADE-CERQual (CERQual) involves an assessment of each individual review finding in terms of four components: (1) methodological limitations; (2) coherence (consistency across primary studies), (3) adequacy of data (the degree of richness and quantity of data supporting the review finding); (4) relevance.

The assessments of the four components collectively contribute to an overall assessment of whether the findings from the qualitative evidence synthesis provide a reasonable representation of the phenomenon of interest. The overall confidence in each review finding is then reported as high, moderate, low, or very low confidence.

The overall CERQual assessments for each review finding were made through initial discussion of between two reviewers (LD and IO) and further discussion with FT. This provided the opportunity to discuss and clearly describe the rationale behind each assessment and was part of the iterative and reflective process of formulating the review findings [42].

The summary of each review finding and indicators of confidence in each review finding are included in Supplementary Appendix: Table 5.

## Findings

### Outcome of study selection

**Fig. 1:**
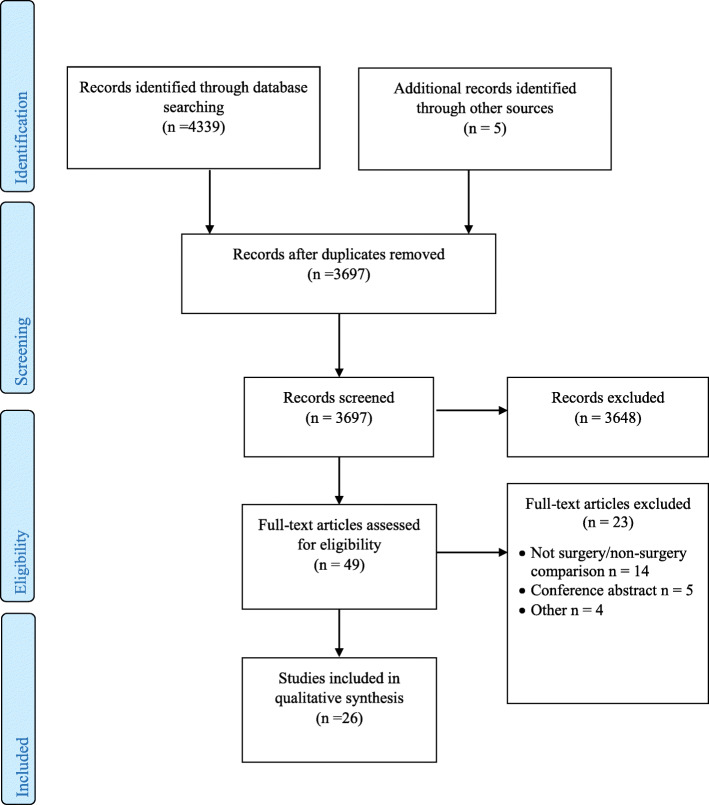
This figure is the PRISMA flow diagram

In total, 4339 titles, 3697 abstracts, and 49 full texts of potentially relevant studies were screened (Fig. 1). Of the 49 potential studies, 23 did not meet the study aims and were excluded. Reasons for excluding the studies included not a surgery/non-surgery comparison (*n* = 14) [43–56], conference abstract only (*n* = 4) [57–60] and protocol/methods paper (*n* = 5) [61–65]. Twenty six studies [66–91] were included in the review. Twenty one studies (21/26) explored experiences within or associated with the context of a single RCT [66–84, 87, 88], whilst 5/26 studies provided a synthesis of results from multiple RCTs [85, 86, 89–91]. A summary of literature search and outcome is presented in the PRISMA flowchart diagram (Fig. 1) [92].

### Characteristics of included studies

Table 3 provides an overview of the 26 included studies, and Table 4 provides details of the characteristics of the studies [66–91] including author, year of publication, country, study aim, sample size, data collection, analytic approach and participants. The studies explored the experience of patients and healthcare professionals. Eleven of the 26 studies included experiences of both patients and healthcare professionals, six included experiences of healthcare professionals only and nine explored the experience of patients only. The majority of trials were conducted in the UK (24/26), and oncology was the most frequently explored clinical speciality (20/26). The focus of the majority of the studies (24/26) were related to the challenges of recruitment [62, 66–75, 77, 78, 80–90]; however, two studies explored reasons for non-compliance to treatment allocation (i.e. why patients crossed over from being randomised to the non-surgical arm to surgical management) following randomisation [76, 79].

**Table 3:** Summary of included studies

Characteristics of studies	Number of studies ***n*** = 26
**Country:**	
UK	24 [67–75, 77–91]
Sweden/Finland/Iceland	2 [66, 76]
**Perspective:**	
Healthcare professionals only	6 [68, 73–75, 77, 90, 91]
Patients only	9 [71, 72, 76, 79, 81–84, 88]
Healthcare professionals and patients	11 [66, 67, 69, 70, 78, 80, 85–87, 89]
**Speciality area:**	
Oncology	20 [66–68, 71–73, 75, 78, 83, 85, 86, 89–91]
Orthopaedics	7 [76, 77, 79, 80, 85, 87, 88]
Urology	2 [74, 81]
Ophthalmology	1 [82]
Obstetrics	1 [84]
Ear, Nose, Throat (ENT)	2 [69, 70]
**Types of comparison:**	
Surgery versus no active intervention	1 [66]
Surgery versus medical management**	13 [67, 68, 72–75, 82, 84, 86, 89–91]
Surgery versus deferred surgery**	2 [69, 70]
Surgery versus medical management versus active monitoring**	5 [71, 78, 83, 89–91]
Surgery versus physiotherapy/exercise component**	5 [76, 77, 79, 80, 87]
Surgery versus placebo versus active monitoring	1 [85]
Surgery versus closed contact casting	1 [88]
Surgery versus medical management versus no active intervention	1 [81]
**Data collection:**	
Qualitative interviews only	16 [66, 69, 70, 72, 74–77, 79–81, 83, 84, 88, 90, 91]
Mixed qualitative methods**	5 [67, 68, 73, 80, 85, 86]
Focus groups	1 [82]
Audio recordings of recruitment appointments	4 [71, 78, 87, 89]

* Includes more than one paper reporting on the same trial

**Mixed qualitative methods, interviews combined with the following: audio recordings of recruitment appointments, focus groups

**Table 4:** Characteristics of studies included in the QES

Reference (study, country)	Study aim/objectives	Stage of trial	Sample	Interventions	Data collection and analysis
1. Bill-Axelson et al (2009) [66] (Sweden, Finland, Iceland)	To understand attitudes to the randomisation process among patients and clinicians in the hope of rendering the process more acceptable for these stakeholders in future.	2-8 years following randomisation	Nine patients with early prostate cancer (*n* = 9, 5 participants and 4 non-participants) and randomising clinicians (*n* = 5)	Radical prostatectomy/watchful waiting	Semi-structured interviews Content analysis
2. Blazeby et al (2014) [67] (United Kingdom)	To determine the feasibility of a main trial of chemotherapy and surgery vs definitive chemoradiotherapy for localised oesophageal squamous cell carcinoma (SCC).	Pre-trial (Feasibility)	26 paired audio recordings of surgery and oncology appointments and 14 in-depth patient interviews.	Neoadjuvant treatment and surgery/definitive chemoradiotherapy	Audio recordings of surgery and oncology appointments and in-depth patient interviews. Conversation analysis Thematic analysis
3. Hamilton et al (2013) [68] (United Kingdom)	To investigate whether it was feasible to undertake a multicentre, RCT comparing disease-free survival and voice outcomes between laser surgery and radiotherapy and particularly factors affecting recruitment and data collection.	Pre-trial (Feasibility)	Surgeons (*n* = 3) and recruiters (*n* = 3)	Endoscopic excision/Radiotherapy	Semi-structured interviews/focus groups/audio recordings of recruitment encounters Content and thematic analysis
4. McSweeney, et al. (2017) [70] (United Kingdom)	To assess the practicality of the proposed internal pilot and full-scale trial.	Pre-trial (Feasibility)	Participants (*n* = 48), ear, nose and throat (ENT) consultants, 1 ET trainee (registrar), research nurses (*n* = 6), nurse practitioners (*n* = 4), trial manager (*n* = 2)	Tonsillectomy/deferred surgery as conservative management option	In-depth interviews Framework analysis
5. McSweeney (2017) [69]. (United Kingdom)	To determine the impact of recurrent sore throats and tonsillitis in adults and stakeholder views of treatment pathways.	Pre-trial (Feasibility)	Patients (*n* = 15), General Practitioners (*n* = 11), 9 ENT consultants, 1 specialist registrar, 6 research nurses, 4 nurse practitioners, 2 trial managers (*n* = 22)	Tonsillectomy/non-surgical management	In-depth interviews Framework analysis
6. Mills et al (2011) [71] (United Kingdom)	To explore how patients’ treatment preferences were expressed and justified during recruitment to a randomised controlled trial (RCT) and how they influenced participation and treatment decisions.	Main trial (Recruitment)	Participants with localised prostate cancer (*n* = 93)	Radical prostatectomy/ radical conformal radiotherapy/active monitoring	Audio recordings
7. Moynihan, C., et al. (2012) (United Kingdom)	To illuminate problems in the context of randomisation to a randomised controlled trial comparing selective bladder preservation against surgery in muscle invasive bladder cancer	Pre-trial (Feasibility)	Patients (*n* = 24)	Radical surgery (cystectomy) following neoadjuvant chemotherapy/ selective bladder preservation where definitive treatment (radiotherapy or cystectomy) was decided based on response to neoadjuvant chemotherapy	Semi-structured interviews Framework analysis
8. Paramasivan et al (2011) [73] (United Kingdom)	To explore reasons for low recruitment and attempt to improve recruitment rates by implementing changes suggested by qualitative findings.	Pre-trial (Feasibility)	Healthcare professionals (*n* = 9)	Radical surgery (cystectomy) following neoadjuvant chemotherapy/selective bladder preservation where definitive treatment (radiotherapy or cystectomy) was decided based on response to neoadjuvant chemotherapy	Audio recordings Semi-structured interviews Conversation analysis Thematic analysis
9. Skea, Z. C., et al. (2017) [74] (United Kingdom)	To explore trial site staff’s perceptions regarding barriers and facilitators to local recruitment.To identify trial-specific modifiable factors that could enhance the facilitators and remove the barriers to recruitment.	Main trial (Recruitment)	Members of staff from 4 trial sites (*n* = 11) Co-applicant (*n* = 1), Principal Investigators (*n* = 3) Consultant Urologist (*n* = 1), Research Nurses (*n* = 5), Research Assistant (*n* = 1)	Extracorporeal shockwave lithotripsy/ureteroscopic stone retrieval (via surgery)	Semi-structured interviews Thematic analysis using Framework approach
10. Strong et al (2016) [75] (United Kingdom)	To explore how teamwork influences recruitment to a multicentre randomised controlled trial (RCT) involving interventions delivered by different clinical specialties.	Main trial (Recruitment)	Healthcare professionals (*n* = 21)	Surgical (oesophagostomy)/definitive chemoradiotherapy)	Semi-structured interviews Thematic analysis
11. Thorstensson (2009) [76] (Sweden)	To understand patients' views about treatment after acute ACL injury, and to explore why patients crossed over from the training only to the surgical and training treatment arm despite consenting to participate in a trial comparing the two treatments.	Main trial	Patients (*n* = 34)	Arthroscopic surgical reconstruction followed by physiotherapist supervised outpatient training (exercise)/supervised training only.	In-depth interviews Framework approach
12. Zeibland et al (2007) [77] (United Kingdom)	To explore their understanding of the trial purpose and how this understanding had influenced their recruitment procedures and interpretation of the results.	Completion of study	Participating surgeons (*n* = 11)	Intensive functional rehabilitation programme (FRP) with spinal fusion surgery for treatment of chronic low back pain	In-depth interviews Thematic analysis
13. Wade et al (2009) [78] (United Kingdom)	To open the “black box” of what goes on during informed consent appointments in a large multicentre RCT (ProtecT).	Feasibility and main study	Recruitment appointments (*n* = 23) 12 recruitment staff	Radical prostatectomy, radical conformal radiotherapy and active monitoring	Audio recordings Thematic, content and conversation analysis
14. Minns Lowe (2017) [79] (United Kingdom)	To explore why participants recruited within UKUFF (The United Kingdom Rotator Cuff Tear Trial) did not remain within their allocated treatment arm, and explored crossover and decisions about having/declining surgery from the perspective of trial participants.	During study	Participants (*n* = 18)	Arthroscopic rotator cuff repair surgery/open/mini-open rotator cuff repair surgery/Rest then Exercise (RtE)	Semi-structured interviews Interpretative Phenomenological Analysis (IPA)
15. Griffin et al (2016) [80] (United Kingdom)	To understand how to optimise recruitment in a future full RCT of this question. Feasibility study comparing surgery and non-operative care for hip impingement.	Feasibility	Trial Management Group (*n* = 10) and clinicians (*n* = 21) RCT consultations recorded (*n* = 87)	Arthroscopic surgery/physiotherapy	Audio recordings In-depth semi-structured interviews Thematic analysis
16. Brookes 2003 [81] (United Kingdom)	To examine the impact of including a ‘no active intervention’ arm (called conservative management) in a RCT comparing treatments (including surgery) for men with lower urinary tract symptoms related to benign prostatic enlargement.	Main trial	Participants (*n* = 22) Non-participants (*n* = 11)	Transurethral resection of the prostate (TURP)/no active intervention (conservative treatment)/non-contact laser therapy.	In-depth semi-structured interviews Thematic analysis
17. Leighton 2012 [82] (United Kingdom)	To examine the attitudes of patients, who presented with advanced glaucoma in at least one eye, to participation in a randomised prospective trial comparing primary medical treatment with primary surgical treatment for advanced glaucoma.	Pre-trial	5 focus groups (between 4 and 8 participants)	Primary medical treatment/primary surgical treatment	Focus groups Thematic analysis
18. Mills 2003 [83] (United Kingdom)	To explore patients’ perceptions of randomisation and understand the reasons for consenting or refusing randomisation within a controversial trial of treatments for localised prostate cancer.	Main trial	Participants in ProtecT study with localised prostate cancer (*n* = 21)	Radical prostatectomy,/radical conformal radiotherapy/ active monitoring	In-depth interviews Constant comparison
19. Lie, M., et al. (2012) [84] (United Kingdom)	To provide insights into two strands of understanding; firstly, women’s experience of participating in research about abortion and secondly, their experience of participating in a randomised preference trial, thus having implications for the design and conduct of termination of pregnancy clinical trials.	Main trial	Participants (*n* = 30)	Medical/surgical termination of pregnancy	Semi-structured interviews
20. Rooshenas et al (2016) [85] (United Kingdom)	To investigate how clinicians conveyed equipoise during recruitment appointments in ongoing RCTs, with the view to identify practices that supported or hindered equipoise communication.	Main trial	Pragmatic UK based RCTs (*n* = 6) Clinicians recruiting to the RCTs (*n* = 16) Appointments in which these clinicians presented the RCT to trial eligible patients (*n* = 105) 2 trials included a surgical/non-surgical comparison	Trial 1: Arthroscopy with surgical manipulation/Arthroscopy alone/Active monitoring with specialist reassessment Trial 2: Neoadjuvant treatment and surgery/definitive non-surgical treatment	In-depth interviews Audio recordings Thematic and content analysis
21. Paramasivan et al (2015) [86] (United Kingdom)	To systematically investigate, quantify and qualitatively explore the imbalances in the presentation of treatments to patients.	Pre-trial (Feasibility)	RCTs (*n* = 2) 1 trial included in this review Recruitment appointments (*n* = 26) patients (*n* = 16) Staff (*n* = 20)	Chemotherapy plus surgery or radiotherapy	Semi-structured interviews Audio recordings
22. Realpe 2016 [87] (United Kingdom)	To investigate the conduct of recruitment consultations that led to participants agreeing to participate in the pilot trial of arthroscopic surgery for hip impingement compared with non-operative care.	Pre-trial (Feasibility)	Consultations (*n* = 92) Participants (*n* = 60_ (*n* = 34 diagnostic, *n* = 58 recruitment consultations)	Arthroscopic surgery/non-operative care.	Audio recordings Thematic and conversational analysis
23. Keene 2016 [88](United Kingdom)	To investigate patients’ experiences of living with a fractured ankle and experiences of being in the trial.	Main trial	Study participants (*n* = 36)	Closed contact casting/Open Reduction Internal fixation (ORIF)	Interviews
24. Mills et al (2014) [89] (United Kingdom)	To investigate how RCT recruiters reacted to patients’ treatment preferences and to identify key strategies to improve informed decision making and trial recruitment.	Main trial (Recruitment)	RCTs (*n* = 3) 2 trials within this Recruitment appointments (*n* = 103) participants (*n* = 96)	Trial 1: Surgical and non-surgical treatment for cancer Trial 2: Surgery/radiotherapy/active monitoring	Audio recordings
25. Donovan et al (2014) [91] (United Kingdom)	To understand the recruitment process from the perspective of recruiters actively recruiting RCT participants in six pragmatic RCTs and to identify opportunities for interventions to improve recruitment.	Feasibility and main trial	RCTs (*n* = 6) 3 trials included in this review Trial 1: Doctors (*n* = 3) Trial 2: Doctors (*n* = 13)/nurses (*n* = 10) Trial 3: Doctors (*n* = 8)/nurses (*n* = 3)	Trial 1: Surgery/radiotherapy Trial 2: Surgery/radiotherapy/active monitoring Trial 3: (Chemotherapy) surgery/radiotherapy	In-depth Interview Content and thematic analysis
26. Donovan et al (2014) [90] (United Kingdom)	To investigate how doctors considered and experienced the concept of equipoise whilst recruiting patients to RCTs.	Feasibility and main trial	RCTs (*n* = 6) 3 trials included in this review Trial 1: Doctors and nurses (*n* = 6) Trial 2: Doctors and nurses (*n* = 20) Trial 3 Doctors (*n* = 8)	Trial 1: Laser surgery/radiotherapy Trial 2: Surgery/radiotherapy/monitoring Trial 3: (Chemotherapy) surgery/radiotherapy	In-depth Interview Content and thematic analysis

### Quality appraisal

The outcome of the quality appraisal of all the 26 included papers and indicators of confidence in each review findings are shown in the Supplementary Appendix: Table 5.

### Concepts and themes

Eighty-five concepts were identified from the 26 studies. A total of 17 of the 85 concepts did not explore the phenomenon of interest: these were related to aspects of the unique trial or the process of randomisation in general, rather than surgical versus non-surgical comparison. No papers were excluded following the coding process. The 68 remaining concepts explored the phenomenon of interest and were developed through progressive abstraction of the data from the primary studies into five themes.

Five main themes were abstracted from the data: (1) radical choice between treatments, (2) patients’ discomfort with randomisation: I want the best treatment for me as an individual, (3) challenge of equipoise: patients’ a priori preferences for treatment, (4) challenge of equipoise: clinicians’ a priori preferences for treatment and (5) imbalanced presentation of interventions.

The themes are discussed below and illustrated with narrative exemplars from the primary studies. Additional examples are included in the Supplementary Appendix: Table 6.

### Themes

#### ‘Radical choice’ between treatments

In contrast to trials with very similar treatment arms, such as the same surgical procedure with/without minor adjustment, clinicians described how they found it challenging to recruit to trials with a surgical and non-surgical comparison as they felt that patients had a strong preference for a particular treatment, largely because they saw the decision as a ‘radical choice’ between the two treatments [72, 73].

The other trials that I work on that are randomised are usually working say between two different types of chemotherapy (…) and no, I haven’t had problems with patients going into studies like that. I think it’s the fact that it is sort of the radical choice if you like, surgery-no bladder, bladder preservation keep your bladder (Nurse, Recruiter) [73].

Similarly, patients described these marked differences between the interventions in these types of trials as having an impact on their views and decision making regarding treatments.

I felt that they [clinicians] were very keen … to say, ‘It doesn’t matter which method you have; it’ll be fine; that’s why it’s OK to have a random trial because the medical’s like this and the surgical’s like this and really there’s no difference between the two’, however, I always kind of felt myself there is a difference between the two; sort of obviously, physically, and secondly, psychologically. I think it does have an impact (Patient) [84].

Some patients expressed clear preference towards one treatment or the other and clinicians’ described this strength of patients’ preference for particular treatments as a major challenge to recruitment in trials with this type of comparison [68, 73, 74, 80].

Clinicians indicated that although some patients accepted that treatments could potentially provide an equally effective outcome, the physical and psychological differences of undergoing a particular treatment, influenced patients’ views towards treatments and trial participation [73, 74].

Either people want it [the cancer] out desperately, don’t want to talk about anything unless it’s out, they want rid of it. Or the group of patients that really doesn’t pander to the idea of a big operation and want to hang on to their bladder, they want to be normal. And so I find a lot of patients come along, even if they haven’t been influenced by somebody else, even if the trial has been put to them beautifully, they usually have some kind of preference based on what happens to them rather than on the effects or the efficacy of the treatment, and I think that’s the biggest problem from the patient’s point of view (Oncologist, Recruiter) [73].

There were four factors related to differences between interventions compared in these types of trials influenced patients’ preferences for treatment:

- Level of invasiveness—surgery cannot be undone

Clinicians felt that differences in the degree of invasiveness between the surgical and non-surgical interventions influenced patients’ preferences for treatment, making the recruitment discussion challenging. Some patients declined trial participation because they felt that if the same results could be achieved without an operation, it was preferable to start with the non-surgical treatment. Surgery could be considered later as an option if there was no improvement with non-surgical management [72, 76].

You can’t undo surgery, but if you have training and don't get well you can always have surgery (Patient) [76].

Preference for the less-invasive option was linked to some patients’ anxiety about surgery [74, 79, 80, 82, 89], with surgery viewed as the point of last resort [80, 82].

A lot of patients seem a lot keener on the lithotripsy (non-invasive treatment for kidney stones) because obviously it’s a much less invasive procedure… we do always say to them, “Well, look. If you have these…you can have up to three treatments … three treatments and if all three of those fail then you will end up having (surgery) anyway", but generally patients are much keener to try the less invasive procedure first, which is understandable (Research Assistant) [74].

(b) Level of potential risk: Is surgery worth the risks?

Differences in the level of potential risk associated with surgery compared to non-surgical treatment, and the implications of those risks [79, 82], influenced patients’ preference towards particular treatments in trials with this type of comparison.

Patient: I’m definitely veering towards the monitoring side of things, because why have all those additional complications, the potential for them… I’ve got a good quality of life and I would like it to continue..... Research nurse: So what would be your worry with surgery? Patient:…With surgery there could be complications… the catheter and impotence [89].

Patients’ views of the potential risk of undergoing surgery and implications of the risks varied depending on the condition or injury being evaluated. Greater duration or severity of symptoms influenced patients towards more invasive treatments, affecting their willingness to participate in a randomsied trial [69, 70, 74, 80].

..I’d be anxious to have the surgery sooner because I’ve been suffering since I was young...to wait even more and to miss more time off work, no I really think it’s time that they come out (Patient) [69].

Some patients considered the potential risks associated with surgery to be ‘worth it’ if it enabled the patient to continue with an activity that was important to them [76].

I imagine the risk of OA increases with surgery. It might be worth the risk if you think you'll have an additional 10 years with football, because it's a long period of your life. If you have OA later, you might be prepared to deal with it, because you had 10 more years with football (Patient) [76].

Differences in the overall strength of patients’ preference for either surgical or non-surgical interventions varied between the trials. For example, the potential risk of blindness from glaucoma surgery (although seen to be low) was identified as a factor underlying strength of patients’ preference for non-surgical treatment in a feasibility study for a trial evaluating surgical or medical management for advanced glaucoma [82].

I think it’s difficult to explain to people that maybe surgery is better, because you could lose your eyesight… in the operation. I don’t know whether that’s happened a lot or not. But if you’re still on the drops, and your eyesight’s going gradually, it might be years before you end up in the same situation that the operation [might create immediately], you know… (Patient) [82].

(iii) Mechanism of effect is clear with surgical treatment, I am confident it will provide a definitive treatment

Comparing interventions with fundamentally different treatment mechanisms also influenced patients’ and clinicians’ willingness to participate in trials with a surgical and non-surgical comparison. Clinicians described how they felt patients were more accepting of the surgical option as they could intuitively understand the effects of surgery, the mechanism of something being repaired or removed. Patients described surgery as providing a cure or ‘fixing’ a problem [80, 86], and viewed surgery as providing a definite treatment, a sense of finality of having received or undergone the treatment [88].

Surgery rids the prostate and therefore rids the cancer. I would be worried about it spreading if I had active monitoring (Patient) [71].

..it’s the best way really to get you on your feet quickly because it’s so fixed and you can’t do damage you know following the op….life’s too short to spend months and months and months around the house worrying about whether or not the bone has knitted properly but I’ve no idea if that is the case…Yes, a quick fix. The way surgeons you know are always saying I fixed you up. You’ll be fine now (Patient) [88].

Non-surgical treatment was not always viewed by patients with as much confidence in being able to provide a definitive treatment to their problem because it was not clear by what mechanism non-surgical treatment alone could repair the damage, correct and provide a ‘fix’ or ‘cure’ [67, 80]. Some patients indicated that they would be uncomfortable with the uncertainty linked to undergoing non-surgical treatment and considered whether the injury was healing or whether they were wasting time before the problem was resolved with surgery [88].

Some patients viewed non-surgical care as a “provisional solution or as a first step treatment plan” [67, 80].

Physio may improve a bit, but I can’t see how physio can resolve the problem if it is hip impingement. It can tear again if I get active, and if I’ve got arthritis, I don’t see how it can get better (Patient) [80].

Patients’ lack of confidence in non-surgical treatment being able to provide a definitive treatment not only affected patients’ willingness to participate in a trial, but was also identified as the reason why some patients randomised to the non-surgical arm did not comply with their treatment allocation, and subsequently underwent surgery [76, 79]. Some patients randomised to non-surgical treatment, considered whether undergoing subsequent surgery would provide a cure, or enable better levels of function to reach full potential, as highlighted in the example below [76, 79].

Training was good, it helped me cope at home, to get stronger and dare more. But I reached the line where I felt good but not 100%. I have been somewhat better, but I want to achieve the last bit. It may get worse as well, they've told me that, even though the chances are quite good. 90–95% get well from surgery, that's quite a lot (Patient, pre-surgery group) [76].

(iv) Disparity between treatment pathways

Marked differences between aspects of the treatment pathways in trials with this type of comparison also influenced patients towards certain treatments. Unlike trials of similar comparison (e.g. two surgical procedures), where interventions are delivered by the same clinical speciality with for example similar waiting times for procedures and level of patient input, patients viewed practical or logistical differences between the trial arms as influencing their decisions around willingness to participate. For example, a more complex treatment process or longer waiting times before being able to start treatment influenced patients’ views towards treatments [68, 73, 74, 79].

Unfortunately, in this facility, sometimes the surgical option is not as quick as…the lithotripsy option. You can see that actually influencing people’s decisions, which they can have the earliest (Research nurse) [74].

With some trial comparisons, patients viewed surgical treatment as more convenient. Whereas the non-surgical treatment was seen as more involved, required more effort or was impractical, for example because of socioeconomic factors such as time off work [68, 79, 80, 85].

[The patient] was with his daughter and… said, very clearly, “I’m not having radiotherapy” because of the travelling every day, because of his family… he wanted something that was in and out… (Recruiter) [68].

#### Patients’ discomfort with randomisation: I want the best treatment for me as an individual?

This theme describes how, although patients accepted the importance of research, they felt uncomfortable if decisions about their individual treatment were left to chance through randomisation, particularly when there were such differences between the treatment comparisons. Some patients indicated that they felt individual factors such as age and extent of problem should be considered in decision making about the best treatment for them as an individual, and felt that a particular treatment might be more suitable than the other [80, 83].

Well I think [random treatment allocation] is quite dodgy. You’d have thought that when you come down to a particular individual their particular circumstance like their age, like the extent of the cancer, like the degree of dispersion of the cancer, like the level of the PSA [prostate-specific antigen], I mean all those individual factors you’d have thought would have some impact on the decision over the treatment…. Wouldn’t you want to identify with the doctor the best treatment for you as an individual? (Patient) [83].

Although some patients found the uncertainty between the different treatments made it easier to decide whether to participate [84], others felt uncomfortable with the loss of ‘control’ over decision making and the departure from a more traditional doctor-patient relationship.

In my head you’ve now firmed everything up … by electing to stay in the study I’m not in control. I’ve always been in control of everything and that is why I wish to opt out of the study and go for regular monitoring. I don’t think at this stage I need [further information] cos I have made up my mind. I’m quite happy that it’s monitoring.” (Patient) [71].

Patients questioned the need for randomisation and how the trial undermined the role of the clinician [80, 83].

I just do what (my consultant) says and that’s it. I would sooner rely on what (my consultant) is telling me. Therefore that’s the route I’m going down, I’m not interested in pros and cons, I’m relying on him to do, err. I’m just interested in, he’s the expert, he’s the one I’ll put my faith in (Patient) [82].

#### Challenge of equipoise: patients’ a priori preferences for treatment

This theme describes clinicians’ challenge of being able to reconcile patients’ a priori views so that they would consider trial participation [68, 73, 74, 80]. Clinicians considered this more challenging in trials where interventions are markedly different to each other and where there is potential for patients to have strong preferences for particular interventions.

Yeah, and the other thing we’re up against… is that patients have this absolute fixation… they have a fixation that somehow laser’s magic right? And I can’t debunk that however much I try (Surgeon 1, interview) [68].

Some recruiting clinicians were reluctant or found it uncomfortable to challenge patients’ expressed preferences for treatment and would accept them without exploring whether or not they were based on accurate information [68, 73, 74, 78, 85, 89, 90].

(Challenging patient’s preferences) depends very much on the patient. If I feel that they’re the sort of person who could cope with the uncertainty, then I’ll say to them, ‘we’re not going to know unless research is done’…… if they absolutely want the bladder removed, you’re not going to be able to sway them, and I don’t think it would be ethical to try to be honest. If they’re adamant, no they want it over with, they want it out, they want it gone, then I don’t think it’s ethical to push, because if it wasn’t the trial, they wouldn’t be having radiotherapy pushed on them anyway, and I don’t feel it’s ethical to push them any further (Nurse, Recruiter) [73].

Challenging patients’ preferences for treatment was viewed by some clinicians as potentially coercive [85], particularly with the elevated risks associated with surgery compared with non-surgical treatment. Some clinicians felt that patients may be ‘rushed into’ a surgical procedure because of participation in the trial.

There are significant risks in people who undertake surgery compared to those that don’t, that’s where it is awkward because these two treatments are so very different (Clinician) [80].

Some clinicians felt that if patients had previously had some form of non-surgical management, such as physiotherapy, this might contribute to patients’ reluctance to undergo further conservative care [76, 79, 80].

Patients come when [they have] already had a course of physiotherapy, that is patients have often come [after] non-operative treatments, and they look at you as if you are mad if you say ‘I want to send you back to physiotherapy for more treatment’ [80].

Exploring individual views about the trial or treatment process and tailoring the information provided to address patient concerns was discussed as a potential strategy to help balance patients’ views about treatments, and enable patients to make an informed choice regarding trial participation [66, 71, 78, 80, 89].

In this situation it’s important to make them understand that the alternatives are equivalent and if a patient preferred surgery I stressed the risks with surgery. The point is to get the patient neutral, not until then is he fully informed and at the same level as current knowledge [66].

#### Challenge of equipoise: clinicians’ a priori preferences for treatment

Clinicians conflict between their willingness to participate and support research and their own individual preferences towards certain treatments for particular patients was regarded as a barrier to trial recruitment. The influence of strong clinical speciality convictions was especially evident in this type of trial comparison.

Clinicians could justify participation through a sense of community equipoise, that is, the collective uncertainty in the clinical community about which treatment is best, due to lack of robust evidence [85, 90]. However, their views about the optimal management of patients on an individual level (“individual equipoise”) sometimes made it difficult to recruit certain individuals.

I’ve not found it [equipoise] personally difficult in concept, because I’ve been absolutely convinced that we don’t know which is the best treatment and although surgery may be a better procedure for cancer cure it has such a high impact that a small number of people might benefit and it’s quite a few who are paying a heavy price to get that. So I’ve not really had any difficulty in that. I think where it’s got difficult, I would say is, in a group of patients that intuitively I feel might do better with active treatment. I know intellectually there’s no evidence about treatment in particular sub-groups, but ... there’s little sub-groups where I’ve found it more difficult than others, and that’s inevitable, I think (Clinician) [91].

Clinicians’ strong preferences for particular treatments or in relation to specific patient groups were described in the studies as contributing to the lack of eligible participants. Patient-related factors such as severity of symptoms, age and specific disease presentation influenced the development of clinicians’ preferences for treatment [66, 68, 80, 85, 90, 91].

My bias is that in a younger person, [intervention 1] probably is a better treatment … Rather than putting them into trial, I think what I’d like to do is give them [intervention 1] up-front (Clinician) [91].

Some clinicians viewed the balance of current evidence to favour one treatment over another, or their clinical experience influenced their strong preferences for certain treatments [80, 85]. This was regarded as more problematic in RCTs evaluating treatments that were already established in clinical practice where clinicians had developed strong views about particular treatments [85, 86].

I spent 20 years learning how to treat patients with certain problems and I’ve gradually learned over the years which ones I can help and which ones I’m less effective in helping (Surgeon) [80].

It is the case though that historically because we’ve had such a strong surgical lead to the MDT (Multi-Disciplinary Team meeting), we’ve got a long history of surgery for that group of patients (…) I think that it’s not because we think surgery is better, it’s just that we’ve more experience and that it’s been a gold standard here for so long. (Oncologist) [86].

Targeted training and support for recruiting clinicians, which was delivered in a number of trials, increased clinicians’ levels of confidence and comfort with equipoise and ability to recruit [90]. Clinicians described how they were more aware of potential prejudices and how they felt more comfortable to challenge their own assumptions [91].

I found the [training] meeting [about trial] fascinating. Because you don’t realise all the prejudices that you do have until you talk to other people about it and you talk about a trial like this and you realise that you are making lots of assumptions, and the main assumption is that as a surgeon you’re doing good. Do you know what I mean? If you get rid of something, you’re doing some good. But you’ve got to critically evaluate that. It’s hard. It’s hard to be honest and say, “I’m doing all this work, slaving away and actually, have I achieved anything?” (Clinician) [91].

#### Imbalanced presentation of interventions

Clinicians’ presentation of the surgical and non-surgical interventions during the recruitment discussion was described as being imbalanced, influencing patients’ views towards treatments and willingness to participate in a trial. There was also a sense that varying involvement and enthusiasm towards the trial by the different clinical specialities influenced patients’ expectations and preferences towards particular treatments.

The terminology that clinicians used to describe treatments was considered to be loaded with meaning or imbalanced, and this affected how patients viewed the treatments [73, 80, 85, 86]. For example in trials comparing surgery and radiotherapy, surgery was described by clinicians using definitive terms such as ‘cure’, ‘kills the cancer’, ‘gold standard’, ‘physically removes’ or ‘cuts it away’ whereas radiotherapy was described more tentatively as ‘ having a chance of killing the tumour’, ‘tries to kill it’, ‘shrinks the tumour’, ‘may be able to treat the tumour’ or as an option for those who did not want an operation [73, 85, 86]. Clinicians’ descriptors of treatment influenced patients’ interpretations, not only on the likelihood of effectiveness, but also on the acceptability of the treatment process. For example, the surgical process was described as ‘nice and simple’ [68], a quicker answer to your health problem [85], whereas non-surgical treatment was described as being ‘a bit more labour intensive’ and ‘a bit further for you to travel’ [68].

Clinician: [after describing definite beneficial effects of treatment x]: However, if you use [treatment y], well, potentially you can get the health benefits that you want, but it does require a lot of input from you.

Patient: Looking at what you told us, [treatment x] would be the best option for me, because it’s a quick answer to my health problems [85].

Variable ‘buy in’ from the clinicians from the different clinical specialities involved in the trial, led some recruiting teams to discuss whether both treatments were presented with equal enthusiasm [75]. Differences in the level of engagement with the study, with some clinicians very committed and others “indifferent” or even “antagonist to it”, created difficulties as patients developed strong preferences for one arm or the other [73, 74].

Some people, urologists especially, in some centres are not behind the trial at all, and will not put patients in towards it, so some centres I know haven’t recruited because there’s a problem with the urologist. Clinicians in general are doing what they think is best, so surgeons are very defensive of cystectomy because they’ve done lots of them, and they think that that’s the best thing to do, and some of them haven’t accepted that the best thing to do is put patients into SPARE [Selective bladder Preservation Against Radical Excision (cystectomy) in muscle invasive bladder cancer] (Investigator) [73].

Some trial teams felt that the order in which patients saw clinicians would create imbalance and influence expectations and the development of preferences towards treatments [73, 75].

Depends who’s spoken (laughs lightly) to them first, I mean if the surgeons have spoken to them first, they generally think that surgery is the best option because it’s been discussed by a surgeon and to a surgeon that is the best option. If they’ve spoken to an oncologist or myself, if they speak to me, they tend to be more open-minded about it, because I present both sides. It’s quite difficult to get people onto a trial saying, ‘hey let’s see if it’s alright to not have surgery’, when the surgeons are saying, ‘well I’d have surgery’. It’s not that (surgeon) is anti-trial or anything else, because he’s not... he’s very pro trials, but his fundamental belief is that if you’ve got a bladder cancer you should have your bladder taken out (Nurse, Recruiter) [73].

Studies in which recruitment discussions were audio recorded described how surgery was often presented at greater length and more favourably than either choosing conservative care or participating in an RCT [67, 80, 87]. Clinicians described the challenge of presenting a treatment arm that is not as familiar to them [67, 77, 80].

Several studies explored ways to create a more balanced approach to recruitment, for example: changing the order that treatments are presented; having combined clinical speciality consultations or multi-disciplinary team meetings to reduce professional barriers; involving another member of the research team better placed to introduce the study to potential patients [75, 87].

P13: You almost want the patients informed and consented and entered into the trial by a non-oncologist and non-surgeons. You want somebody who knows about both treatments, very well and in detail, knows about potential complications but also is removed from the frontline, so that they can impart as much information as possible in an unbiased way (Consultant surgeon) [75].

Other trials redrafted patient information sheets or advice given to recruiters to provide information in a more balanced way [73, 87].

### Conceptual model

**Fig. 2:**
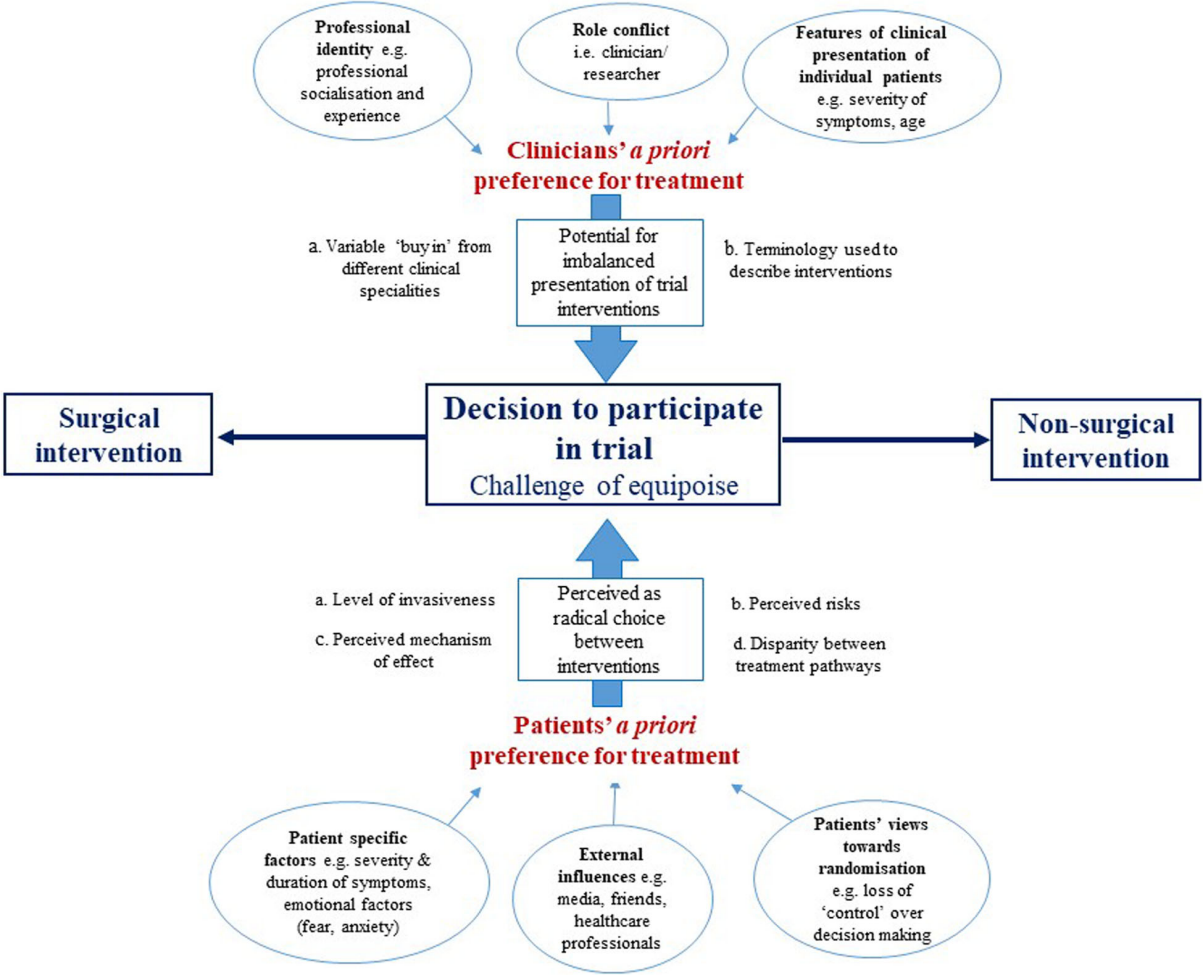
Conceptual model for QES

Figure 2 illustrates the model we developed which integrated the findings from this review to derive new insights and help understand the challenges of conducting trials with a surgical and non-surgical comparison [20].

The model is based on the concept of a seesaw, centred on the decision of whether to participate in a clinical trial. The impact of several factors from both a patient and clinician perspective could potentially tip the balance towards strong preferences for either surgery or the non-surgical intervention making trial participation potentially challenging. The model highlights the potential difficulty both patients and clinicians may have achieving a position of equipoise in these types of trials where the intervention comparators can be so diverse.

For example, depending on the trial comparison the degree of differences between surgical and non-surgical interventions in the level of invasiveness and perceived risks, understanding of perceived mechanism of effect and any disparity between treatment pathways may shift the balance and strength of patients’ views towards one treatment or another. Severity and duration of symptoms, emotional factors (fear, anxiety), external influences (media, health care professionals) and views towards randomisation and may also shift the balance of patients’ views towards one treatment or another.

Similarly, the influence of strong clinical specialty convictions may be particularly challenging in these types of trial comparisons where interventions compared are typically delivered by different clinical specialties. Clinicians’ a priori preference for treatment may influence their decision to participate or how information about the trial is portrayed, potentially influencing the balance of patients’ views towards treatments.

The analogy of the seesaw in the decision-making process in trials of this type of comparison highlights the possibility of potentially addressing the imbalance in some factors, and improving conduct and participation.

## Discussion

The aim of this review was to conduct a synthesis of qualitative studies to help us to understand the experiences of patients and healthcare professionals in trials comparing surgical and non-surgical interventions and to identify challenges to their design and conduct. Findings highlight factors related to the marked dichotomy between the surgical and non-surgical interventions influenced patients’ and clinicians’ views towards treatment. The findings of this review increase our understanding of why patients and clinicians may find equipoise more challenging in these types of trials and consequently may make recruitment potentially more difficult compared to other trial comparisons.

Patients treatment preferences are identified as one of the main challenges to recruitment in clinical trials [93], and these are known to be particularly problematic in trials comparing surgical and non-surgical interventions [5–7]. In the context of RCTs, the development of patients’ preferences is known to be complex [91, 94]. Bower et al. [94] in their conceptual model of the development of preferences in randomised trials in general, define preference as the “difference in the perceived desirability of two (or more) interventions within an RCT”. The concepts of *utility* ‘a measure of satisfaction gained from the consumption of a good or service, such as health care’ [95] and *attitude* ‘a disposition to respond favourably or unfavourably to an object, person, institution or event’ [96] were central to their model [94]. Bower et al. conceptualise patients as having a particular ‘strength’ of preference from a ‘slight preference which has little substantive importance, through to large preferences which have a major influence on behaviour’ [94]. The findings of this current review add to the growing body of evidence [11, 16–19], by identifying factors that may contribute to the strength of patients’ preference for a surgical or non-surgical intervention in trials with this specific type of comparison, and which may contribute to reported difficulties with recruitment [5–7].

The marked contrast between the invasiveness and irreversibility associated with the surgical and non-surgical procedures contributed to patients’ reluctance to participate in trials with this type of comparison. Previous studies have discussed how surgical procedures can raise particular emotions (e.g. fear, anxiety) and surgical interventions can be associated with particularly high negative value [94, 97]. The importance of considering emotional influences on patient decision making in trials was discussed by Bower et al. [94] and can be particularly salient in trials of this type of comparison, where the magnitude of difference between the interventions is so great. The findings of this review suggest that to some patients, surgery is viewed as a ‘point of last resort’ or, as also discussed by Sibai et al. [97], the option to consider if there are no improvements with non-surgical care. The potential for a patients’ preference for treatment to be dynamic has also been discussed in previous studies [18, 94, 98], decision making on trial participation may therefore relate to the time point in the treatment pathway a patient is approached. For example, in a trial comparing an elective surgical procedure with non-surgical management, a patient may have previously undergone some form of non-surgical treatment, which may affect their views towards treatment and trial participation. Understanding the specific treatment pathway and patients’ views on treatments at a pre-trial stage could be hugely beneficial to identify factors that may affect the development of preferences and influence recruitment.

The clear difference in the level of potential risk associated with surgery compared to non-surgical interventions was also identified as influencing patients’ preference towards treatments. However, specific factors, such as a patients’ particular diagnosis or condition, duration and severity of symptoms could also be seen to affect a patients’ perception of the risk associated with treatments. Previous studies have discussed the importance of considering potential participants’ health state (life-threatening conditions, long-term conditions), and trajectory of their condition (established or recently diagnosed conditions) during recruitment to clinical trials [18, 19, 98]. For example, a trial of two surgical procedures, urethrotomy and urethroplasty, for recurrent urethral stricture by Whybrow et al. [98] found that patients decisions regarding willingness to participate was seen to be related to a patients’ symptom progression. Patients either felt the condition was ‘too slight to consider a serious operation or too severe not too’ [98]. In this trial, one of the treatments was more invasive and viewed by patients as potentially being curative, whereas the other procedure was relatively straightforward operation but considered more as symptom palliation [98]. Whybrow et al. considered that there may only be a ‘particular window of opportunity in which patients would be willing to accept either procedure’. Exploring the strength of patients’ preference for particular interventions, and factors underpinning any preferences, prior to trial conduct, could provide valuable information to trial teams and enable issues specific to a particular trial to be identified and addressed.

The findings of this review also indicate that explanations of a condition or injury as a mechanical problem with a mechanical fix were seen to be understood more intuitively by patients and influenced strength of patients’ preference towards surgery. Surgery was viewed by patients as providing a definitive treatment to the problem, whereas patients expressed difficulty in understanding how a non-surgical intervention could achieve a similar outcome. Patients lack of confidence in the expected effectiveness of non-surgical treatments compared to surgery has also been highlighted in studies exploring reasons for treatment choices made in routine clinical practice [99–101]. Ensuring that both surgical and non-surgical interventions are understood and viewed by patients as comparative, despite the differences between them in mechanism of effect, is important because of the potential impact on patients’ views towards treatments and trial participation. Providing balanced information and addressing patients’ views towards treatments to ensure potential patients are fully informed about treatments, could be beneficial in helping patients make decisions regarding trial participation.

In this review, disparity between the treatment pathways of the surgical and non-surgical interventions also contributed to patients’ views towards the different treatments. A more complex treatment process, differences in level of patient input required, timing of when patients would receive the intervention, and socioeconomic factors, for example, time off work, were seen to contribute to patients’ views towards treatments and trial participation. As discussed by Bower et al. in their conceptual model, ‘process issues’ [102–104] such as financial cost of personal convenience may be equally important to patients when making decisions regarding trial participation [94]. The findings of this review highlight that unlike trials of similar comparisons (e.g. two surgical procedures), aspects of the interventions are more likely to be markedly different in these types of trials, potentially having a greater impact on patients views, and may contribute to why recruiting to these types of trials can be seen as more challenging than those of trials of interventions with similar comparisons [5–7]. Understanding the treatment pathway of the interventions being compared and patients’ views towards treatments could provide valuable information to trial teams and enable issues specific to a particular trial to be identified and addressed.

The findings of this review resonate with previous studies which have demonstrated patients can find the concept of randomisation challenging [11, 19, 105, 106]. As with trials of other types of comparisons (for example, two surgical procedures), patients were uncomfortable with the loss of ‘control’ over decision making and the departure from a more traditional doctor-patient relationship, undermining the role of the clinician. The marked differences in the interventions compared in these types of trials resulted in some patients being reluctant to leave decision about their individual treatment to chance through randomisation. The findings of this review suggest that patients in these types of trials considered that individual differences in symptom presentation, age, or previous non-surgical care would impact on decisions made over their treatment, making one option more suitable than the other.

Discussing trial participation with potential patients who have strong preferences for treatment was also seen to be particularly challenging for recruiting clinicians in trials of this type of comparison. The stark differences between the interventions were considered by some clinicians to make the process more difficult than for example trials of two similar surgical procedures [90, 91]. Challenging patients’ preferences for treatment was viewed by some clinicians as potentially coercive [85], particularly with the elevated risks associated with surgery compared with non-surgical treatment. Some clinicians felt that patients may be ‘rushed into’ a surgical procedure because of participation in the trial [80]. It could be argued that in cases where strong preferences for treatment exist, patients should not be approached to participate and should receive their treatment of choice [71, 107]. However, studies have shown that exploration of patients’ initial views about treatments is sometimes based on misunderstanding of scientific evidence [71]. Exploring patients’ preferences and providing patients with evidence-based information, therefore, could be seen as an important and integral part of the information exchange necessary to enable patients to make informed decisions not only about trial participation, but also about their own care [71]. Training recruiting clinicians to explore patients’ treatment preferences has shown to facilitate recruitment in trials with such diverse comparisons [71, 108, 109]. Understanding factors underpinning patients preferences for treatments and providing trial-specific training for recruiting staff [67, 73, 75] may help to ensure a more balanced approach to recruitment in these types of trials and facilitate recruitment.

The findings of this review also highlight the potential conflict clinicians can have in their dual role as a clinician and participating in research. Clinicians’ strong preferences for treatment are a well-recognised challenge to the conduct of clinical trials, potentially having a detrimental impact on recruitment [11, 29, 110, 111]. The additional influence of strong clinical speciality convictions was identified in this review as being especially evident in trials with this type of comparison. Professional preferences of the different specialities involved in these types of trials may result in more complex issues concerning social roles, power and authority influencing recruitment [112]. Clinicians’ experience, knowledge and views on available evidence, both RCT and non-RCT, influenced preferences towards treatments and were seen to affect their position of equipoise. These findings reflect those of previous studies [11, 113, 114], where the difficulties clinicians can experience in a dual role of clinical care and research are discussed. Ingrained values, skills and knowledge that clinicians develop over time through ‘intensive professional socialisation’ [114] are considered to make it difficult for clinicians to totally separate from the care and welfare of patients when involved in research [113, 114]. In this review, clinicians’ preference for treatment was seen to be potentially more influential where trials challenged established treatments, which may reflect the potential for stronger professional views towards the treatment of a patient population to develop over time through experience. Alternative approaches to randomisation may be required in cases where equipoise make recruitment particularly problematic. Understanding clinicians’ perspectives at a pre-trial stage could identify equipoise issues and help develop effective and targeted training to support recruitment and engagement from all specialities.

The findings of this review also resonate with previous studies [11, 19] which suggest that the way treatments are discussed, and the terminology used by recruiting clinicians has the potential to be imbalanced, contributing to patients views towards treatment and trial participation. As trials evaluating surgical versus non-surgical interventions typically involve more than one clinical speciality, the difficulties were seen to be potentially more problematic. The potential for asymmetry in clinicians’ delivery of information about surgical and non-surgical treatments to patients has also been identified in studies of clinical practice [115–117]. A study by Hudak et al. of orthopaedic surgery consultations showed that when surgeons discussed surgery versus non-surgical options, surgery was portrayed as having a ‘special, privileged status relative to other options’ [116]. The structure of surgeons’ treatment recommendations were seen to potentially shape patients’ expectations, whether for surgery or some alternative. In trials with surgical and non-surgical comparators, where there is potential for imbalance in how interventions are portrayed, consideration should be given to the terminology used to describe the intervention comparators and how this may influence patients’ perceptions of the interventions.

## Limitations

There are some limitations to this review. Only published literature was included, other sources such as unpublished dissertations were not included. Similarly, studies not published in the English language were excluded, meaning that relevant publications may have been omitted. The majority of the trials were of elective surgery and therefore some of the aspects may not be applicable to the trials in the more acute setting such as fracture management, where there may be differences in the consent process. In addition, as outlined in the summary of included studies table (Table 3), the majority of the studies were from an oncology speciality and trials were largely conducted in the UK. Several of the papers discussed findings from the same trial, limiting the number of different trials included. However, a range of specialties and comparisons were included which provided a valuable in-depth insight into expectancies, beyond that of the individual studies.

The majority of the published literature were of studies that explored challenges to recruitment in trials of this type of comparison. Further primary research could be conducted to explore areas identified as challenging in these types of trials where knowledge is lacking, such as non-compliance and reasons for differential loss to follow-up.

The coding of the papers was only conducted by one reviewer (LD). However, two reviewers (LD and IO) read each paper thoroughly to become familiar with the studies. In addition, several reviewers (LD, IO, FT, DB) were involved in discussion throughout the process as part of the iterative and reflective process of formulating the review findings (45).

Finally, there is no agreed way to determine confidence in QES findings which are an interpretation based on abstraction. Further studies to explore the utility of GRADE-CERqual for conceptual reviews such as meta-ethnography would be useful.

## Conclusion

Factors related to the diverse nature of the surgical and non-surgical comparison were identified in this review as influencing patients’ and clinicians’ views towards treatments. These factors could result in difficulties for patients and clinicians to achieve equipoise when considering trial participation and consequently making recruitment challenging.

The characteristics identified in this research can provide trialists with areas of exploration (by qualitative feasibility studies) before they start recruitment to a trial comparing surgical and non-surgical interventions. Understanding the potential challenges will facilitate more efficient design choices and benefit the delivery of such trials. Future research could also aim to assess the impact of implementing pre-trial qualitative feasibility work and subsequent interventions on recruitment.

## Supplementary Information

**Additional file 1.** Supplementary Appendix.

## Data Availability

The datasets used and/or analysed during the current study are available from the corresponding author on reasonable request.

## Abbreviations

CASP: Critical appraisal skills programme
QES: Qualitative evidence synthesis
RCT: Randomised controlled trial
SoQF: Summary of Qualitative Findings
