# Supplementary material for: The challenge of equipoise in trials with a surgical and non-surgical comparison: a qualitative synthesis using meta-ethnography
Source: Trials. 2021 Oct 7;22:678. doi: 10.1186/s13063-021-05403-5 (PMC8495989; doi:10.1186/s13063-021-05403-5)
Supplement: Supplementary file 1 — Additional file 1. Supplementary Appendix. [file 13063_2021_5403_MOESM1_ESM.zip › SupplementaryAppend_CASP checklist.docx]

### Supplementary Appendix: Critical Appraisal Skills Programme (CASP) checklist

Q1. Was there a clear statement of the aims of the research?

Q2. Is qualitative methodology appropriate?

Q3. Was the research design appropriate to address the aims of the research?

Q4. Was the recruitment strategy appropriate to the aims of the research?

Q5. Was the data collected in a way that addressed the research issue?

Q6. Has the relationship between researcher and participants been adequately considered?

Q7. Have ethical issues been taken into consideration?

Q8. Was the data analysis sufficiently rigorous?

Q9. Is there a clear statement of findings?

Q10. How valuable is the research?

1=No, 2=Can’t tell from details in paper, 3=Yes.

| Reference  (Study, country) | Q1. | Q2. | Q3. | Q4. | Q5. | Q6. | Q7. | Q8. | Q9. | Q10. |
| --- | --- | --- | --- | --- | --- | --- | --- | --- | --- | --- |
| 1. Bill-Axelson et al (2009) ^69^ | 3 | 3 | 3 | 3 | 3 | 2 | 2 | 3 | 3 | 3 |
| 1. Blazeby et al (2014) ^70^ | 3 | 3 | 3 | 3 | 3 | 2 | 2 | 3 | 3 | 3 |
| 1. Hamilton et al (2013) ^71^ | 3 | 3 | 3 | 3 | 3 | 2 | 2 | 3 | 3 | 3 |
| 1. McSweeney, et al. (2017) ^72^ | 3 | 3 | 3 | 3 | 3 | 2 | 2 | 3 | 3 | 3 |
| 1. McSweeney (2017).^73^ | 3 | 3 | 3 | 3 | 3 | 2 | 2 | 3 | 3 | 3 |
| 1. Mills et al (2011) ^74^ | 3 | 3 | 3 | 3 | 3 | 2 | 2 | 3 | 3 | 3 |
| 1. Moynihan, C., et al. (2012) ^75^ | 3 | 3 | 3 | 3 | 3 | 2 | 2 | 3 | 3 | 3 |
| 1. Paramasivan et al (2011) ^76^ | 3 | 3 | 3 | 3 | 3 | 2 | 2 | 3 | 3 | 3 |
| 1. Skea, Z. C., et al. (2017) ^77^ | 3 | 3 | 3 | 3 | 3 | 3 | 2 | 3 | 3 | 3 |
| 1. Strong et al (2016) ^78^ | 3 | 3 | 3 | 3 | 3 | 2 | 2 | 3 | 3 | 3 |
| 1. Thorstensson (2009) ^79^ | 3 | 3 | 3 | 3 | 3 | 2 | 2 | 3 | 3 | 3 |
| 1. Ziebland et al (2007) ^80^ | 3 | 3 | 3 | 3 | 3 | 2 | 2 | 3 | 3 | 3 |
| 1. Wade et al (2009) ^81^ | 3 | 3 | 3 | 3 | 3 | 2 | 2 | 3 | 3 | 3 |
| 1. Minns Lowe (2017) ^82^ | 3 | 3 | 3 | 3 | 3 | 2 | 3 | 3 | 3 | 3 |
| 1. Griffin et al (2016) ^83^ | 3 | 3 | 3 | 3 | 3 | 2 | 2 | 3 | 3 | 3 |
| 1. Brookes (2003) ^84^ | 3 | 3 | 3 | 3 | 3 | 2 | 2 | 3 | 3 | 3 |
| 1. Leighton (2012) ^85^ | 3 | 3 | 3 | 3 | 3 | 2 | 2 | 3 | 3 | 3 |
| 1. Mills (2003) ^86^ | 3 | 3 | 3 | 3 | 3 | 2 | 2 | 3 | 3 | 3 |
| 1. Lie, M., et al. (2012) ^87^ | 3 | 3 | 3 | 3 | 3 | 3 | 3 | 3 | 3 | 3 |
| 1. Rooshenas et al (2016) ^88^ | 3 | 3 | 3 | 3 | 3 | 3 | 2 | 3 | 3 | 3 |
| 1. Paramasivan et al (2015) ^89^ | 3 | 3 | 3 | 3 | 3 | 2 | 3 | 3 | 3 | 3 |
| 1. Realpe (2016) ^90^ | 3 | 3 | 3 | 3 | 3 | 2 | 3 | 3 | 3 | 2 |
| 1. Keene (2016) ^91^ | 3 | 3 | 3 | 3 | 3 | 2 | 2 | 2 | 3 | 2 |
| 1. Mills et al (2014) ^92^ | 3 | 3 | 3 | 3 | 3 | 2 | 3 | 3 | 3 | 3 |
| 1. Donovan et al (2014) ^93^ | 3 | 3 | 3 | 3 | 3 | 2 | 2 | 3 | 3 | 3 |
| 1. Donovan et al (2014) ^94^ | 3 | 3 | 3 | 3 | 3 | 2 | 3 | 3 | 3 | 3 |
