# Supplementary material for: The challenge of equipoise in trials with a surgical and non-surgical comparison: a qualitative synthesis using meta-ethnography
Source: Trials. 2021 Oct 7;22:678. doi: 10.1186/s13063-021-05403-5 (PMC8495989; doi:10.1186/s13063-021-05403-5)
Supplement: Supplementary file 1 — Additional file 1. Supplementary Appendix. [file 13063_2021_5403_MOESM1_ESM.zip › SupplementaryAppend_Search strategy for QES.docx]

### Supplementary appendix. Search strategy for QES

Example search strategy:

1. Qualitative evidence synthesis (QES) search strategy

| Example search syntax for MEDLINE |
| --- |
| (((“semi-structured”[TIAB] OR semistructured[TIAB] OR unstructured[TIAB] OR informal[TIAB] OR “in-depth”[TIAB] OR indepth[TIAB] OR “face-to-face”[TIAB] OR structured[TIAB] OR guide[TIAB] OR guides[TIAB]) AND (interview*[TIAB] OR discussion*[TIAB] OR questionnaire*[TIAB])) OR (“focus group”[TIAB] OR “focus groups”[TIAB] OR qualitative[TIAB] OR ethnograph*[TIAB] OR fieldwork[TIAB] OR “field work”[TIAB] OR “key informant”[TIAB])) OR “interviews as topic”[Mesh] OR “focus groups”[Mesh] OR narration[Mesh] OR qualitative research[Mesh] OR "personal narratives as topic"[Mesh] OR (theme[TIAB] OR thematic[TIAB]) OR "ethnological research"[TIAB] OR phenomenol*[TIAB] OR "grounded theory"[TIAB] OR "grounded study"[TIAB] OR "grounded studies"[TIAB]OR "grounded research"[TIAB] OR "grounded analysis"[TIAB] OR "grounded analyses"[TIAB] OR "life story"[TIAB] OR "life stories"[TIAB] OR emic[TIAB] OR etic[TIAB] OR hermeneutics[TIAB] OR heuristic*[TIAB] OR semiotic[TIAB] OR "data saturation"[TIAB] OR "participant observation"[TIAB] OR "action research"[TIAB] OR "cooperative inquiry"[TIAB] OR "co-operative inquiry"[TIAB] OR "field study"[TIAB] OR "field studies"[TIAB] OR "field research"[TIAB] OR "theoretical sample"[TIAB] OR "theoretical samples"[TIAB] OR "theoretical sampling"[TIAB] OR "purposive sampling"[TIAB] "purposive sample"[TIAB] "purposive samples"[TIAB] OR "lived experience"[TIAB] OR "lived experiences""purposive sampling"[TIAB]  OR "content analysis"[TIAB] OR discourse[TIAB] OR "narrative analysis"[TIAB] OR heidegger*[TIAB] OR colaizzi[TIAB] OR spiegelberg[TIAB] OR "van manen*"[TIAB] OR "van kaam"[TIAB] OR "merleau ponty"[TIAB] OR husserl*[TIAB] OR Foucault[TIAB] or Corbin[TIAB] OR Strauss[TIAB] OR Glaser[TIAB] |
| Randomized controlled trial.pt. OR Controlled clinical trial.pt. OR Randomized.ab. OR Clinical trials as topic OR Randomly.ab. OR Trial.ti. |
