# Supplementary material for: The challenge of equipoise in trials with a surgical and non-surgical comparison: a qualitative synthesis using meta-ethnography
Source: Trials. 2021 Oct 7;22:678. doi: 10.1186/s13063-021-05403-5 (PMC8495989; doi:10.1186/s13063-021-05403-5)
Supplement: Supplementary file 1 — Additional file 1. Supplementary Appendix. [file 13063_2021_5403_MOESM1_ESM.zip › SupplementaryAppend_Table 5.docx]

Table 5: Summary of Qualitative Findings (SoQF) table

| **Review Finding** | **Studies Contributing to the Review Finding** | **Assessment of Methodological Limitations** (details of CASP checklist included in Supplementary Appendix) | **Assessment of Relevance** | **Assessment of Coherence** | **Assessment of Adequacy** | **Overall CERQual assessment of Confidence** | **Explanation of judgement** |
| --- | --- | --- | --- | --- | --- | --- | --- |
| 1. Radical choice between treatments | 13/26 studies ([70-72](#_ENREF_70), [74](#_ENREF_74), [76](#_ENREF_76), [77](#_ENREF_77), [79](#_ENREF_79), [82-85](#_ENREF_82), [87](#_ENREF_87), [91](#_ENREF_91)) | Minor methodological concerns (not clear in a number of studies whether the relationship between the researcher and participants was adequately considered) | Moderate concerns. All included studies were exploring issues related to trials where there was a surgical and non-surgical comparison. Some of the concepts in some studies were related to aspects of the individual trials and did not fit the analysis. | Moderate concerns. Overall the majority of studies included in this review are oncology studies conducted in the UK, however a range of studies from different specialities contributed to this finding. Data consistent within and across all studies. Data reflects the variation in the types of interventions compared in the different studies and highlights differing views depending on the comparison. | Moderate concerns about adequacy (13 studies contributed to this review finding providing moderately rich data overall). | Moderate confidence | The finding was graded as moderate confidence because of minor concerns regarding methodological limitations and moderate concerns regarding adequacy, relevance and coherence. |
| 1. Patients’ discomfort with randomisation: I want the best treatment for me as an individual | 7/26 studies ([69](#_ENREF_69), [70](#_ENREF_70), [74](#_ENREF_74), [75](#_ENREF_75), [83](#_ENREF_83), [85](#_ENREF_85), [124](#_ENREF_124)) | Minor methodological concerns (not clear in a number of studies whether the relationship between the researcher and participants was adequately considered) | Moderate concerns. All included studies were exploring issues related to trials where there was a surgical and non-surgical comparison. Some of the concepts in some studies were related to aspects of the individual trials and did not fit the analysis. | Moderate concerns.  Overall the majority of studies that contributed to this finding were oncology studies conducted in the UK. Data was well supported by the details in the underlying studies. | Moderate concerns about adequacy (7 studies contributed to this review finding providing moderately rich data overall). | Moderate confidence | The finding was graded as moderate confidence because of minor concerns regarding methodological limitations and moderate concerns regarding adequacy, relevance and coherence. |
| 1. Challenge of equipoise: Clinicians’ *a priori* preferences for treatment. | 6/26 studies ([69](#_ENREF_69), [71](#_ENREF_71), [83](#_ENREF_83), [88](#_ENREF_88), [93](#_ENREF_93), [94](#_ENREF_94)) | Minor methodological concerns (not clear in a number of studies whether the relationship between the researcher and participants was adequately considered) | Moderate concerns. All included studies were exploring issues related to trials where there was a surgical and non-surgical comparison. Some concepts in some studies were related to aspects of the individual trials and did not fit the analysis. | Moderate concerns. Overall, the majority of studies that contributed to this finding were oncology and orthopaedic studies conducted in the UK. Data was consistent within and across all studies. | Moderate concerns about adequacy (6 studies contributed to this review finding providing moderately rich data overall). | Moderate confidence | The finding was graded as moderate confidence because of minor concerns regarding methodological limitations and moderate concerns regarding adequacy, relevance and coherence. |
| 1. Imbalanced presentation of intervention. | 9/26 studies  ([70](#_ENREF_70), [76-78](#_ENREF_76), [83](#_ENREF_83), [88-90](#_ENREF_88), [93](#_ENREF_93)) | Minor methodological concerns (not clear in a number of studies whether the relationship between the researcher and participants was adequately considered) | Moderate concerns. All included studies were exploring issues related to trials where there was a surgical and non-surgical comparison. Some concepts in some studies were related to aspects of the individual trials and did not fit the analysis. | Moderate concerns. Overall, the majority of studies that contributed to this finding were oncology studies conducted in the UK. Data was well supported by the details in the underlying studies. | Moderate concerns about adequacy (9 studies contributed to this review finding providing moderately rich data overall). | Moderate confidence | The finding was graded as moderate confidence because of minor concerns regarding methodological limitations and moderate concerns regarding adequacy relevance and coherence. |
| 1. Challenge of equipoise: Patients’ *a priori* preferences for treatment. | 8/26 studies  ([69-71](#_ENREF_69), [76](#_ENREF_76), [81](#_ENREF_81), [83](#_ENREF_83), [92](#_ENREF_92), [94](#_ENREF_94)) | Minor methodological concerns (not clear in a number of studies whether the relationship between the researcher and participants was adequately considered) | Moderate concerns. All included studies were exploring issues related to trials where there was a surgical and non-surgical comparison. Some concepts in some studies were related to aspects of the individual trials and did not fit the analysis. | Moderate concerns. Overall, the majority of studies that contributed to this finding were oncology studies conducted in the UK. Data was well supported by the details in the underlying studies. | Moderate concerns about adequacy (8 studies contributed to this review finding providing moderately rich data overall). | Moderate confidence | The finding was graded as moderate confidence because of minor concerns regarding methodological limitations and moderate concerns regarding adequacy relevance and coherence. |
