# Supplementary material for: The challenge of equipoise in trials with a surgical and non-surgical comparison: a qualitative synthesis using meta-ethnography
Source: Trials. 2021 Oct 7;22:678. doi: 10.1186/s13063-021-05403-5 (PMC8495989; doi:10.1186/s13063-021-05403-5)
Supplement: Supplementary file 1 — Additional file 1. Supplementary Appendix. [file 13063_2021_5403_MOESM1_ESM.zip › SupplementaryAppend_Table 6.docx]

Table 6: Final themes, studies supporting themes and additional narrative exemplars from the primary studies.

| Theme | Examples of narrative from primary study |
| --- | --- |
| 1. Radical choice between treatments.   Primary studies supporting this category:([70-72](#_ENREF_70), [74](#_ENREF_74), [76](#_ENREF_76), [77](#_ENREF_77), [79](#_ENREF_79), [82-85](#_ENREF_82), [87](#_ENREF_87), [91](#_ENREF_91)) | *….Oncologist: Ok. What, in particular puts you off the surgery? Patient: I don’t think I’m particularly worried about surgery, as such,… - if the [non-surgical option] can do the same thing without the cutting that’s my only reason, really [in picking it]. Oncologist: Ok. That’s fair enough’ (*[*89*](#_ENREF_89)*).*  *It’s a very difficult decision and there isn’t a right and a wrong answer and some people have very clear ideas about what they want to do, whether it should be an operation, they want to have it cut out. Other people, the thought of the operation is just so frightening they opt for the radiotherapy treatment. But for us, we don’t know which of these treatments is - is better (Oncologist).(*[*89*](#_ENREF_89)*)*  *So having chemo and radiotherapy, there’s no guarantee with any of it, but in my mind, you’re just trying to shrink the tumour and there is no guarantee that you will do it with that, but with surgery you’re physically cutting it out (Patient)(*[*89*](#_ENREF_89)*).*  *Surgery for most people has got to be the last option, now if they can sort it out with physio, and after 12 months of physio doesn’t work, OK let’s do the surgery, I think that’s acceptable to me, I won’t have a problem with that, it is information that I’d like to have (Patient).(*[*83*](#_ENREF_83)*)*  *Thing is, when you’ve got (some sight), even if you’ve lost a little sight, you’ve got something. But if the operation wasn’t successful, you might end up with nothing’ (Patient).(*[*85*](#_ENREF_85)*)*  *If they have the symptoms the same as somebody else and the other person’s having surgery to correct it, the person who’s having physio would think ‘how can it be corrected without the surgery?’ (Patient).(*[*83*](#_ENREF_83)*)*  *“My shoulder had improved, I thought shall I go (for surgery) and I thought…the surgery will fix it…I wanted a cure because I knew somebody who had surgery and it cured it completely” (Patient). (*[*82*](#_ENREF_82)*)*  *‘If you come in with a tumour… but you’ve got more than a ninety percent chance of curing this whatever we do, ninety ﬁve if it’s really small… you’ve got a life, you’ve got kids to manage, you’ve got a job to manage and you’ve got all the rest of it to manage, what are you going to choose?’ (Surgeon)(*[*71*](#_ENREF_71)*).*  *Because you have to be out work and in crutches, and it would have implications for me for childcare if I have it, I don’t know how immobile you’d be (Patient)(*[*83*](#_ENREF_83)*).* |
| 1. Patients’ discomfort with randomisation: I want the best treatment for me as an individual.   Primary studies supporting this category:([69](#_ENREF_69), [70](#_ENREF_70), [74](#_ENREF_74), [75](#_ENREF_75), [83](#_ENREF_83), [85](#_ENREF_85), [124](#_ENREF_124)) | *I don’t necessarily agree [that clinicians don’t know the best treatment]. One would be more suitable to one patient and something else to another…. I’m not really in favour of it [randomization]. I can understand why you would rather have it randomized, so that nothing else affects it, but I think all patients are probably different and that difference, the state of their health and so on, is different (Patient). (*[*86*](#_ENREF_86)*)*  *I think it ought to be dependent on your particular situation rather than on a 50: 50 toss of a coin which way you went. I’m sure some issues, and some hip injuries, are probably more suited to one route rather than other (Patient) (*[*83*](#_ENREF_83)*).*  *“(...) but the only thing I don’t understand is when you are pulled out of the computer.. .that’s when the problem started (I withdrew) ’you will be picked out at random’ was what they said...and I had no more control.. .I got out.” (Patient). (*[*75*](#_ENREF_75)*)*  *What he said it was either the knife or the radiotherapy or this wait and see business, which would be, if I would agree, by computer random choice and I said, “Well, yes” because I’ve got in back of my mind that whoever’s programmed that computer has got to have some kind of medical knowledge because obviously someone whose got a very large cancer, which could cause death straight away or within a few months, I can’t imagine his name being down on a wait-and-see basis. What I’m trying to say, there’s got to be a level somewhere where they can say, “Yes, we’ll wait,” “No, we can’t wait.” I’m hoping’, I’m putting’ me faith in it (Patient). (*[*85*](#_ENREF_85)*)*  *I understood that [treatments were equally effective], but I just ﬁnd it difﬁcult to deal with a random approach to anything. To feel that this very important decision, which is genuinely a decision about the possibility of life or death at some point in the future, being down to chance, I ﬁnd that difﬁcult to accept. I ought to be able to do better than that. I ought to work it out, the one that is most appropriate for me. I think, well, one of the three is going to be better than the other two for me (Patient).(*[*86*](#_ENREF_86)*)*    *Interviewer: Did he explain why treatment was randomized? Mr. Williams: Well, yeah, because I mean they didn’t know enough about it and so they obviously wanted so many people to agree and wanted an even number as possible, I suppose, of people. Obviously in research they want to compare each one, don’t they. But I thought, well, I want the one that is suitable for me.(*[*86*](#_ENREF_86)*)*  *It is not ethical if it’s random [. . .] It makes you wonder 50 : 50 if you’re just going to be put in one or the other without looking at how your symptoms are or how you are, it sounds worrying (Patient). (*[*83*](#_ENREF_83)*)* |
| 1. Challenge of equipoise: Patients’ a priori preferences for treatment.   Primary studies supporting this category:([69-71](#_ENREF_69), [76](#_ENREF_76), [81](#_ENREF_81), [83](#_ENREF_83), [92](#_ENREF_92), [94](#_ENREF_94)) | *‘Research nurse: Have you had a chance to look through the literature you were given in the clinic about the different treatment options?*  *Patient: Yeah I did....I mean I’d like to go for [minimally invasive surgical option].*  *Research nurse: Okay that’s fine. I’ll let [recruiter’s] secretary know that’. (*[*92*](#_ENREF_92)*)*  *Patient: Because from what I can gather it’s not that bad, it hasn’t spread anywhere else. So the chances are this, this [non-surgical option] could cure it. Oncologist: That’s absolutely right Patient: You know if it had gone anywhere else I’d, I would have said straightaway well go for that [surgical option]…. Oncologist: Yep, yeah I understand that it’s a worrying time isn’t it. So you would rather go for the [non-surgical option]? Patient: I think that’s the best thing to do. Oncologist: Yep ok, that’s absolutely fine….I think that’s the right thing to do actually’. (*[*92*](#_ENREF_92)*)* |
| 1. Challenge of equipoise: Clinicians’ a priori preferences for treatment   Primary studies supporting this category:([69](#_ENREF_69), [71](#_ENREF_71), [83](#_ENREF_83), [88](#_ENREF_88), [93](#_ENREF_93), [94](#_ENREF_94)) | *Hmm, I have to say, I mean being honest, with the more elderly patients with more co-morbidities, diabetes, I tend to go straight for [procedure x] on those patients (in routine practice). Hmm, just because they’ve got a limited life expectancy. In the younger patients I get with the less co-morbidities, I certainly have a preference for [procedure y] (Clinician).(*[*88*](#_ENREF_88)*)*  *The literature by and large shows 80% of the people get good results, as far as symptoms go with surgical treatment. There is no evidence anywhere that conservative treatment will work (Clinician). (*[*83*](#_ENREF_83)*)*  *“I wouldn’t be involved with the trial because it wouldn’t be ethical if I had a strong view that this wasn’t right … But, there are a subset of younger men with more aggressive tumours where I find that really quite difficult.”(Clinician).(*[*93*](#_ENREF_93)*)*  *I think the radiotherapists should believe in radiotherapy and I think surgeons believe in surgery. And I think that’s probably pretty normal actually. To tell you the truth, I believe in surgery because I’m a surgeon. And I think what we’ll ﬁnd out from the study is whether the radiotherapy treats as well as we do (Clinician). (*[*93*](#_ENREF_93)*)*  *As far I am concerned, there isn’t a position of equipoise for consent on this, because there is enough data in the literature, which admittedly, isn’t high-quality data, particularly with cam impingement, we get good results with surgery in terms of symptom relief (Clinician). (*[*83*](#_ENREF_83)*)*  *There’s a proportion of patients who will say to me, ‘‘What do you think doctor?’’ And in that situation, I think my gut feeling is important. I always tell them. I wouldn’t have become a surgeon if I thought another form of therapy was the best form of therapy, would I? So my preference does matter to some patients but, you know, it’s important for them in my opinion to also go and talk to the radiotherapist who, presumably, will be more passionate about their treatment (Clinician).(*[*94*](#_ENREF_94)*)* |
| 1. Imbalanced presentation of interventions.   Primary studies supporting this category:([70](#_ENREF_70), [76-78](#_ENREF_76), [83](#_ENREF_83), [88-90](#_ENREF_88), [93](#_ENREF_93)) | *I think the main problem of the trial was who saw them first. If the oncologist saw them first then they would be able to lay out their stall first – with the pluses and the minuses and then if they were seen again – in a few days’ time by the surgeons then foremost in their mind would be what the oncologist had to offer and we (as surgeons) would then have to, in inverted commas, compete with that. Whereas, if it happened the other way round we would then at least get our information across on a blank canvas. I think that’s the difficult thing with two very different treatments with two very different side-effect and complication profiles (Surgeon).(*[*78*](#_ENREF_78)*)*  *‘So you come in, you go to theatre, you have your operation, you come back to the ward, you wake up and then you toddle off home and you’re all done. And that’s nice and simple… Sounds great, doesn’t it?’*  *‘The radiotherapy which you discuss with doctor X is a bit more intensive, that’s a bit more, you know, labour intensive. So you’d have to go into X hospital for that, which is a bit further for you to travel. But you would be going over there every day for six weeks, but you’re only there about ﬁfteen twenty minutes, so it’s quite quick so although it sounds a lot, six weeks of treatment’ (Recruiter, recruitment appointment).(*[*71*](#_ENREF_71)*)*  *You almost want the patients informed and consented and entered into the trial by a non-oncologist and non-surgeons. You want somebody who knows about both treatments, very well and in detail, knows about potential complications but also is removed from the frontline, so that they can impart as much information as possible in an unbiased way (Surgeon).(*[*78*](#_ENREF_78)*)*  *In my view the patient gets a bias towards surgery from the start (...) The patient tends to hang on to the first thing, so even if the patient gets to a radiotherapist (...) they quite often say, “oh yes, I’ll have surgery” even if they get to the radiotherapist. By the time they get to me for what is supposed to be the pros and cons and the discussions around randomisation, they quite often have things set in their mind (Oncologist, Recruiter). (*[*76*](#_ENREF_76)*)*  *Some of my colleagues in the unit would want to operate on absolutely everything they had the opportunity to operate on, and so might not emphasise the chemoradiotherapy bit quite as strongly. (Surgeon). (*[*78*](#_ENREF_78)*)*  *But of course the surgeon is obviously more biased to surgery and the oncologist is biased to that treatment. I think subconsciously the way they talk to them… It may be something even more subtle than in words that they are saying. (Research nurse). (*[*78*](#_ENREF_78)*)*  *Clinician: The first is [treatment x] to remove the cancer to give us the chance of cure.*  *Clinician [later]: [Treatment y] is designed to shrink down the tumour and try and kill it off.*  *Patient relative: The thing that’s worrying me if she has [treatment y if that doesn’t take it away but if she has [treatment x] then it’s gone.(*[*88*](#_ENREF_88)*)*  *There was someone who used to work here who used to say ‘well I can cut away your tumour or they can shrink it with radiotherapy’, so the word you use matters a lot because ‘shrink’ gives the impression that you’re not going to get rid of it, whereas to cut it out means you’ve taken it away, gives you the impression that you’re definitely cured (Oncologist).(*[*89*](#_ENREF_89)*)*  *I normally talk about the fact that we need to do chemotherapy ... and that the surgery is the ‘gold standard’ way of managing the cancer and that there is a possibility of the selective bladder preservation possibly being an alternative, but we need to confirm that in the trial and that’s why we’re doing a trial (Oncologist, Recruiter). They’re either having the ‘gold standard’ treatment or an experimental treatment (Nurse, Recruiter).(*[*76*](#_ENREF_76)*)*  *We have a large trial portfolio and we try to have ‘buy-in’ from the whole team to a clinical trial so the clinical trials that we do are agreed by the whole team then, not done in isolation. And the MDT decision will be to offer a clinical trial. That’s one of the reasons why we’ve been very successful in terms of actually putting people into trials because there is ‘buy-in’ from the whole team to do clinical research. So, I think it was an MDT decision that they were eligible for the study and we went from there. (Oncologist).(*[*78*](#_ENREF_78)*)*  *We can certainly stir up and reinforce a patient’s bias very easily with a throw away comment like the aim of the surgery is to cut out your cancer and that could ruin everything from the point of view of a balanced randomised trial (Surgeon).(*[*89*](#_ENREF_89)*)* |
