# Supplementary figures and images for: The challenge of equipoise in trials with a surgical and non-surgical comparison: a qualitative synthesis using meta-ethnography
Source: Trials. 2021 Oct 7;22:678. doi: 10.1186/s13063-021-05403-5 (PMC8495989; doi:10.1186/s13063-021-05403-5)

Supplementary Appendix: Example of thematic map


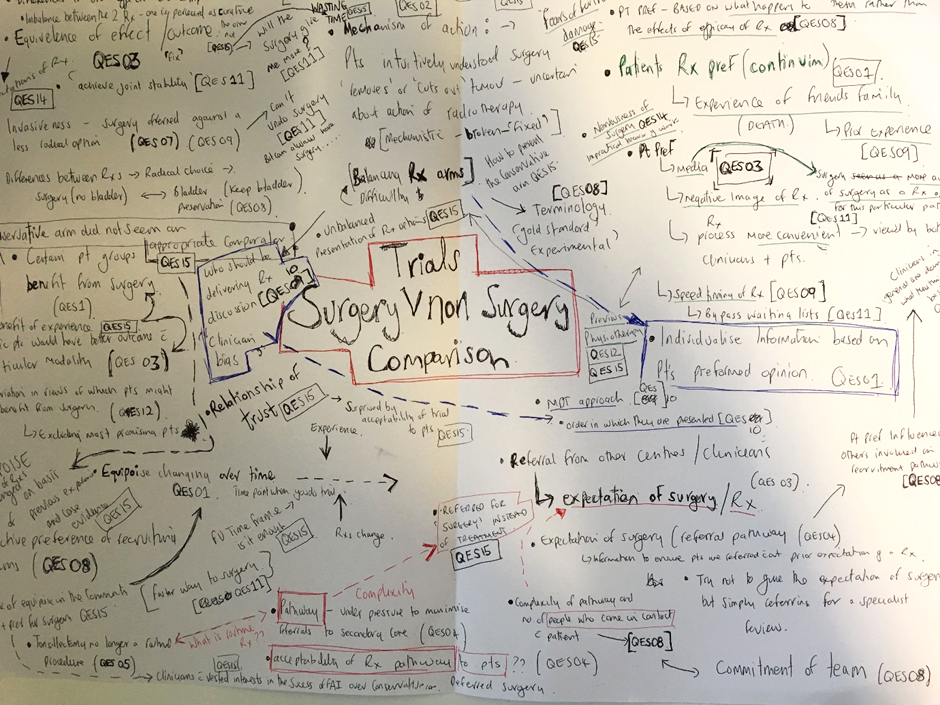

Supplement: Supplementary file 1 — Additional file 1. Supplementary Appendix. [file 13063_2021_5403_MOESM1_ESM.zip › SupplementaryApp_ExampleofThematicMap.docx]
